# Dietary supplementation of Eucommia leaf extract to growing-finishing pigs alters muscle metabolism and improves meat quality

**DOI:** 10.5713/ab.23.0220

**Published:** 2023-11-01

**Authors:** Zhenglei Shen, Chuxin Liu, Chuangye Deng, Qiuping Guo, Fengna Li, Qingwu W. Shen

**Affiliations:** 1 College of Animal Science and Technology, Southwest University, Chongqing 400715, China; 2 College of Food Science and Technology, Hunan Agricultural University, Changsha, Hunan 410128, China; 3 Institute of Subtropical Agriculture, Chinese Academy of Sciences, Changsha, Hunan 410125, China

**Keywords:** *Eucommia ulmoides*, Glycolysis, Meat Quality, Metabolomics, Pre-slaughter Transport

## Abstract

**Objective:**

The objective of this study was to investigate the influence of dietary supplementation of *Eucommia ulmoides* leaf extract (ELE) on muscle metabolism and meat quality of pigs with and without pre-slaughter transportation.

**Methods:**

In a 43-day feeding experiment, a total of 160 pigs with an initial body weight 60.00±2.00 kg were randomly assigned into four groups in a completely randomized design with 10 replicates. Pigs in groups A and C were fed a basal diet and pigs in groups B and D were fed a basal diet supplemented with 0.5% ELE. Pigs were slaughtered with (group B and D) or without (group A and C) pre-slaughter transport. Muscle chemical composition, postmortem glycolysis, meat quality and muscle metabolome were analyzed.

**Results:**

Dietary ELE supplementation had no effect on the proximate composition of porcine muscle, but increased free phenylalanine, proline, citruline, norvaline, and the total free amino acids in muscle. In addition, dietary ELE increased decanoic acid and eicosapentaenoic acid, but decreased heptadecanoic acid, oleic acid, trans-oleic acid, and monounsaturated fatty acids in muscle. Meat quality measurement demonstrated that ELE improved meat water holding capacity and eliminated the negative effects of pre-slaughter transport on meat cooking yield and tenderness. Dietary ELE reduced muscle glycolytic potential, inhibited glycolysis and muscle pH decline in the postmortem conversion of muscle to meat and increased the activity of citrate synthase in muscle. Metabolomics analysis by liquid chromatographic tandem mass spectrometric showed that ELE enhanced muscle energy level, regulated AMP-activated protein kinase (AMPK) signaling, modulated glycogenolysis/glycolysis, and altered the metabolism of carbohydrate, fatty acids, ketone bodies, amino acids, purine, and pyrimidine.

**Conclusion:**

Dietary ELE improved meat quality and alleviated the negative effect of pre-slaughter transport on meat quality by enhancing muscle oxidative metabolism capacity and inhibiting glycolysis in postmortem muscle, which is probably involved its regulation of AMPK.

## INTRODUCTION

With the improvement of living standards, there has been an increasing demand for high-quality meat products. Compared with the autochthonous rustic pig breeds, the pork quality of the imported commercial crossbreeds is being increasingly criticized by consumers in China due to its reduced sensory quality [1,2]. At the same time, genetic selection for lean growth dramatically increased the occurrence of pale, soft, and exudative (PSE) meat [3]. PSE meat is a well-known phenomenon that frequently occurs in pork and poultry. It is primarily caused by abnormal muscle metabolism that occurs after slaughter. In other words, fast and excessive glycolysis in muscle early postmortem (PM) when muscle temperature is high causes a rapid drop of muscle pH and denaturation of protein, which is the cause of PSE syndrome [4,5]. PSE meat has a high drip loss, a low cooking yield, and a tough texture after cooking. For its inferior quality, PSE meat is not liked by both consumers and meat processors. Statistics shows that the average occurrence of PSE in pigs in China is 15% to 30%, which causes a huge economical loss every year [6].

Besides genetic issues, pre- and post-slaughter handling and slaughter operations have a significant impact on PM meat quality development [7]. Inappropriate pre-slaughter handling, such as antemortem stress induced by transport, is a major factor causing PSE meat. Previous studies show that pre-slaughter transportation accelerates the depletion of ATP in porcine muscle, causing the body to be in a lower energy state. A lower energy state leads to the activation of AMP-activated protein kinase (AMPK), which in turn accelerates glycolysis and increases the incidence of PSE meat [8]. In addition, oxidative stress is induced in animals during transportation, which breaks down the animals’ homeostatic conditions before slaughter. In this process, an excessive amount of reactive oxidants is generated in muscle tissue and stress hormones are released into the bloodstream, causing abnormal rise in heart rate, blood pressure, and body temperature. As pre-slaughter handling, including loading, transportation, lairage, mixing of strange animals, and stunning which all induce stresses either psychologically or physically and consequently adversely affect meat quality, is unavoidable in practice, nutritional strategy and on-farm handling seems to be a feasible method to reduce the negative effect of antemortem stress and to improve meat quality.

Natural plant products are widely used as feed additives to ameliorate stress during the production process, ensuring good animal welfare and animal product quality. *Eucommia ulmoides* is a plant that is commonly used in traditional Chinese medicine. It is rich in bioactive compounds, such as alkaloids, glycosides, and phenols, with antibacterial, anti-inflammatory and antiparasitic properties. In addition, dietary supplementation of *Eucommia ulmoides* leaf extracts (ELE) has been shown to improve animal growth, antioxidative capacity and intestinal function in weaned piglets and large yellow croaker larvae, and even to enhance the water holding capacity of chicken breast muscle [9–11]. However, its impact on muscle metabolism and pork quality, especially the meat quality from pigs subjected to pre-slaughter stress is not explored.

In this study, we determined the effects of dietary ELE supplementation for 6 weeks on meat quality of pigs subjected to pre-slaughter transport stress and showed that dietary ELE supplementation improved pork quality, partially eliminated the adverse effect of antemortem stress on meat quality development by altering muscle metabolism.

## MATERIALS AND METHODS

All animal procedures were approved by the Protocol Management and Review Committee of Institute of Subtropical Agriculture, Chinese Academy of Science (No. 84 20190045).

### Animals and diets

*Eucommia ulmoides* leaf extract was purchased from Zhangjiajie Hengxing Biotechnology Co., Ltd (Zhangjiajie, China), and the main active ingredients of ELE contains chlorogenic (5%), flavonoids (8%) and polysaccharide (20%).

Totally, 160 crossbred pigs with similar initial body weight (Duroc×Landrace×Yorkshire, DLY, 60.00±2.00 kg) were randomly assigned into four groups, with 10 pens per group and 4 pigs per pen. Pigs in groups A and C were fed a basal diet with 16% crude protein (CP) to meet the nutritional needs for growing-finishing pigs according to the National Research Council (NRC, 2012). Pigs in groups B and D were fed a basal diet supplemented with 0.5% ELE. All diets were isoenergetic and the ingredients and nutritional composition of basal diet were listed in Supplementary Table S1. Pigs had *ad libitum* access to diets and drinking water. The feeding trial was conducted for 43 days.

### Muscle sample collection

At the end of feeding trial, 10 pigs (one pig per pen) were randomly selected from each group and slaughtered at a commercial slaughter plant (Hunan TRS Co., LTD, Zhuzhou, China). Pigs in groups A and B were delivered to the abattoir 12 h prior to slaughter. Pigs in groups C and D were transported for one-hour right before slaughter to stress animals. At 0, 0.75, 4, and 24 h PM, a slice of *Longissimus thoracis* (LT) muscle between the 10th and 11th thoracic vertebrae in the left side of carcass was dissected and snapfrozen in liquid nitrogen for chemical, enzyme activity and metabolomics analysis. One boneless pork loin chop was dissected at 24 h PM from the same location in the right side of carcass for meat quality measurement. Groups A–D were named Control, ELE, T_1 h_, and T_1 h_+ELE.

### Chemical analysis

The moisture, ash, protein, and lipid content in muscle (0 h PM) were analyzed according to GB5009.3-2016 National Food Safety Standard-Determination of Moisture in Foods, GB5009.4-2016 National Food Safety Standard-Determination of Ash in Foods, GB5009.5-2016 National Food Safety Standard-Determination of Protein in Foods and GB5009.6-2016 National Food Safety Standard-Determination of Lipid in Foods, respectively.

Glycogen, glucose, and lactate in muscle were analyzed using commercially available assay kits (Solarbio, Beijing, China) according to the manufacturer’s instruction. Muscle glucose-6-phosphate was measured using an assay kit from Sigma (Sigma, St. Louis, MO, USA). Glycolytic potential (GP) was calculated as GP = 2× ([glycogen]+[glucose]+ [glucose-6-phosphate])+(lactate). The content of glycogen in muscle was calculated as μmol glucose/g muscle. GP was calculated as μmol lactate/g muscle.

The free amino acids (FAA) in muscle were determined by HPLC as in literature [12]. Briefly, FAA in muscle were extracted in pure water. Pre-column derivation of amino acids was conducted using ortho-phthalaldehyde for the primary amino acids and 9-fluorenylmethyloxycarbonyl for the secondary amino acids in 0.4 M borate buffer (pH 10.2). Prepared samples were analyzed using an Agilent 1100 HPLC (Agilent Technology Inc., Waldbronn, Germany) equipped with a VWD detector. A mixed amino acid standard (Sigma, USA) was used for sample quantification [12].

Fatty acids in porcine LT muscle were profiled by gas chromatograph mass spectrometer (GC-MS) as in literature [12]. Briefly, total lipid in muscle was extracted using benzene: petroleum ether (1:1) with glycerol triundecanoate (Nu-Chek Prep Inc., Minnesota, PA, USA) in methanol as internal standard. The lipid extract was added with KOH in methanol for fatty acid methyl esters (FAME) preparation. The FAME were then separated and identified using an Agilent7890-5975 GC-MS (Agilent, Germany). A mixed standard containing 37 FAME (Supelco Inc., Bellefonte, PA, USA) was used as external standards for peak identification and quantification [12].

### Meat quality measurements

Muscle pH was measured using a pH meter (Sartorius Scientific Instruments Co., Ltd., Beijing, China) after homogenization of 0.1 g of muscle in 0.9 mL of 5 mM iodoacetic acid solution. Meat surface color (CIE L*a*b*) of boneless loin chops were measured using a portable colorimeter YS3010 (Shenzhen San’enshi Technology Co., Ltd., Shenzhen, China) as described in literature [13]. Meat drip loss, cooking yield and shear force were measured as previously described [13].

### Enzyme activity analysis

The enzymatic activities of glycogen phosphorylase a (GPa), pyruvate kinase (PK), and citrate synthase (CS) were measured using commercial kits (Solarbio, Beijing, China) according to the manufacturer’s procedure. One unit of GPa activity was defined as one nanomole of NADPH produced from glucose-6-phosphate by glucose-6-phosphate dehydrogenase per minute per gram of muscle tissue. One unit of PK activity was defined as one nanomoles of NADH oxidized per minute per gram of muscle sample. One unit of CS activity was defined as one nanomole of 5′-thionitrobenzoate (TNB) produced from 5,5′-dithiobis-(2-nitrobenzoic acid) (DTNB) per minute per gram of muscle protein. Protein concentration in the supernatant of muscle homogenate was determined by BCA assay.

### Untargeted metabolomic analysis by liquid chromatographic tandem mass spectrometric

Muscle tissues (0 h PM) were pulverized in liquid nitrogen. 25 mg of powdered sample were added with 500 μL of extraction solution (MeOH:ACN:H_2_O, 2:2:1, v/v) containing isotope labeled internal standards, sonicated on ice, and centrifuged at 13,800×g, 4°C for 15 min. The supernatants was collected and passed through a 0.22 μm filter before used for liquid chromatographic tandem mass spectrometric (LC-MS/MS) analysis [14,15].

LC-MS/MS analyses were performed using an UHPLC system (Vanquish; Thermo Fisher Scientific, Waltham, MA, USA) with a Waters ACQUITY UPLC BEH Amide (2.1 mm×50 mm, 1.7 μm) column coupled to Orbitrap Exploris 120 mass spectrometer (Orbitrap MS; Thermo, USA). The mobile phase consisted of 25 mmol/L ammonium acetate and 25 mmol/L ammonia hydroxide in water (pH = 9.75) (A) and acetonitrile (B). The sample loading temperature was 4°C and the injection volume was 2 μL. The Orbitrap Exploris 120 mass spectrometer was used to acquire MS/MS spectra on information-dependent acquisition (IDA) mode in the control of the acquisition software (Xcalibur; Thermo, USA). The electrospray ionization source conditions were sheath gas flow rate 50 Arb, Aux gas flow rate 15 Arb, capillary temperature 320°C, full MS resolution 60000, MS/MS resolution 15000, collision energy SNCE 20/30/40, spray voltage 3.8 kV (positive) or −3.4 kV (negative), respectively.

The raw data were converted to the mzXML format using ProteoWizard and processed for peak detection, extraction, alignment, and integration using an in-house program. Metabolites were annotated with an in-house MS2 database (BiotreeDB). The cutoff for annotation was set at 0.3.

### Statistical analysis

Statistical analysis was performed by a two-way analysis of variance for diet and pre-slaughter transport effects with the software SPSS 25.0 (SPSS Inc., Chicago, IL, USA) and the individual pig (n = 10) as the experimental unit. The means and standard errors of means were calculated for the variables. Differences were considered significant when p<0.05. The graphs were plotted using OriginPro 8 software.

## RESULTS AND DISCUSSION

### Dietary supplementation of ELE altered chemical composition of porcine muscle

*Eucommia ulmoides* is a traditional Chinese herb containing a variety of bioactive compounds, including lignin, iridoids, phenolics, steroids, terpenoids, etc. The bioactive parts of *Eucommia ulmoides*, including leaves, seeds, bark, and staminate flower, are widely used as raw materials for medicine, food and recently for animal feed additives. The leaves and bark of *Eucommia ulmoides* are antiviral and antibacterial, and used in animal feed to reduce the usage of hormones and antibiotics. In addition, several studies have shown that extract from *Eucommia ulmoides* leaf improves the oxidative status in chickens, pigs, lamb, and even type 2 diabetic mice [11,16–18]. Chlorogenic acid-enriched extract from *Eucommia ulmoides* leaf alleviates the adverse effects of heat stress on growth performance and meat quality of broilers [19]. However, the effects of ELE on muscle metabolome in relationship to pork quality, especially the meat quality from pigs subjected to pre-slaughter stress, have not yet been investigated.

In the present study, pigs were dietarily supplemented with 0.5% ELE and subjected to one hour transport before slaughter. As shown in Table 1 to 4, dietary supplementation of ELE had no effect on the proximate composition of porcine muscle as no difference in the content of moisture, ash, CP, and total fat was detected between the control and ELE groups. However, the moisture was significantly lower (p< 0.05) in the muscle from stressed pigs, which could be attributed to the low water holding capacity (Table 4) contributed by antemortem stress.

**Figure 1:**
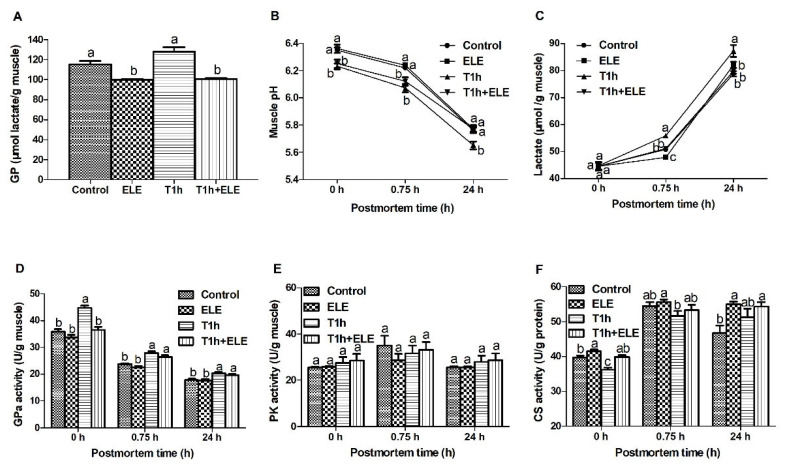
Glycolysis and metabolic enzyme activities in *Longissimus thoracis* muscle of pigs. At the same PM time point, means lacking a common letter differ at p<0.05. n = 10.

Free amino acids in fresh muscle (0 h PM) was determined by HPLC. The result showed that dietary ELE supplementation had an obvious influence on the content of free alanine, phenylalanine, proline, citruline, norvaline, and the total FAA which was significantly higher (p<0.05) in the muscle from pigs supplemented with ELE when compared with the control (Table 2). Citrulline is an intermediate metabolite of the urea cycle, which is produced from ornithine, while norvaline is an inhibitor of arginase, which catalyzes the hydrolysis of arginine to produce urea. The increased concentrations of these two amino acids in muscle reflected that dietary ELE might alter urea metabolism in pigs and likely reduced nitrogen emission through urea cycle, which is probably responsible for the improved growth performance of pigs [18]. Hydroxyproline is an indicator of collagen content. Although no difference in hydroxyproline was detected between the control and ELE pigs, proline was higher in the muscle from ELE pigs. Proline is used for collagen synthesis and hydroxylated to hydroxyproline after incorporation into protein. Some studies have reported that *Eucommia ulmoides* leaves promote collagen synthesis in rats, boilers, and eels [20–22], the modulation of free proline in muscle by *Eucommia ulmoides* leaves detected here could be the mechanism. Phenylalanine is an essential amino acid. It is converted to tyrosine by phenylalanine hydroxylase and then used for hormone synthesis within cells, like dopamine, adrenaline, norepinephrine, thyroxine and so on. The altered phenylalanine content in porcine muscle indicated that dietary ELE might modulate muscle metabolism through the synthesis of hormones, which was supported by the enriched glucagon signaling pathway predicted by metabolomics analysis (Table 6). Glucagon regulates glycogen mobilization to maintain blood glucose concentrations. Indeed, dietary ELE supplementation reduced GP in porcine muscle (Figure 1A), which could influence PM glycolysis in muscle and consequently meat quality. Different to dietary ELE, pre-slaughter transport did not have a significant effect on amino acid metabolism within muscle. Among the determined 23 FAA in porcine muscle, 21 amino acids, along with the total FAA, were not different in concentrations between the two groups supplemented with ELE (ELE vs T_1 h_ +ELE) and the two groups supplemented without ELE (Control vs T_1 h_). Pre-slaughter transport increased (p<0.05) hydroxyproline in the muscle from pigs supplemented with ELE but decreased (p<0.05) the concentration of methionine in all pigs supplemented with or without ELE (Table 2). In summary, all these data showed that dietary ELE had a comprehensive effect on the metabolism of amino acids in porcine muscle. First, dietary ELE increased the concentrations of free citruline and norvaline in porcine muscle, indicating it may improve animal growth performance [18] by modulating urea metabolism and nitrogen emission. Second, dietary ELE increased the content of free phenylalanine in muscle, regulated glucagon signaling and GP in porcine muscle, which may subsequently affect PM glycolysis and meat quality development.

Fatty acid composition within muscle is shown in Table 3. Although the concentration of total fatty acids were not affected by dietary supplementation of ELE, which is consistent with the total lipid content in muscle (Table 1), the content of multiple fatty acids altered in muscle from ELE pigs when compared to the control. Previously, it has been reported that dietary ELE increases short-chain fatty acids in the serum of mice [23]. In agreement with literature, dietary supplementation of ELE increased (p<0.05) the concentration of decanoic acid (C10:0), a relative short but medium-chain fatty acid determined in the present study. In heat-stressed broilers, chlorogenic acid-enriched extract from *Eucommia ulmoides* leaf reduces the content of stearic acid and saturated fatty acids, but increases dihomo-γ-linolenic acid, linoleic acid, linolenic acid, eicosapentaenoic acid, and polyunsaturated fatty acids in breast muscle [19]. Here dietary supplementation of ELE reduced (p<0.05) the content of heptadecanoic acid (C17:0), oleic acid (C18:1, n9c), trans-oleic acid (C18:1, n9t), and monounsaturated fatty acids in the LT muscle of pigs when compared with the control. The content of eicosapentaenoic acid (C20:5, n3c) was determined to be increased (p<0.05) by dietary ELE in porcine muscle. Although it is not statistically different, the content of stearic acid and saturated fatty acids tended to decrease (0.05<p<0.08) in the muscle from pigs supplemented with ELE. In addition, ELE supplementation decreased (p<0.05) the n-6/n-3 ratio. Based on these data, it could be concluded that dietary ELE supplementation was beneficial to human health in terms of the pork fatty acid composition. Pre-slaughter transport had an obvious influence on fatty acids in the muscle from pigs supplemented with ELE. When the two groups of pigs supplemented with ELE were compared, the content of most fatty acids, including C10:0, C14:0, C16:0, C16:1, C18:0, C18:1, C18:2. C20:0, C20:3, C20:4 and C20:5, and the total fatty acids, were significantly decreased (p<0.05) by pre-slaughter transport in the muscle from T_1 h_+ ELE pigs. However, the fatty acid profile in the muscle from pigs without ELE supplementation was not affected by pre-slaughter transport. This may reflect some difference in muscle metabolism between pigs supplemented with and without ELE, indicating that pigs supplemented with ELE relied more on fatty acids and oxidative phosphorylation for energy generation when stressed, which was supported by the higher CS activities (Figure 1F) in ELE pigs. In summary, dietary supplementation of ELE changed the fatty acid profile in porcine muscle and increased fatty acid oxidation in response to stress. As lower GP (Figure 1A) and higher CS activities (Figure 1F) were also determined in the muscle from pigs supplemented with ELE, it can be concluded that dietary ELE supplementation changed muscle metabolism pattern and increased muscle oxidative metabolism capacity, which consequently influenced PM muscle glycolysis and meat quality.

### Dietary supplementation of ELE improved pork quality

The meat quality attributes of porcine LT muscle are listed in Table 4. Pre-slaughter transport has an adverse effect on meat quality, the drip loss of meat from the two groups of pigs transported for one hour before slaughter was significantly higher (p<0.05) than that of the unstressed pigs. The meat from ELE pigs had the lowest drip loss, which was significantly lower (p<0.05) than that of meat from the unstressed control pigs. In addition, the cooking yields of the meat from both the ELE and T_1 h_+ELE groups was higher (p<0.05) than that of the T_1 h_ group, showing that dietary supplementation of ELE increases water holding capacity of meat from antemortem stressed pigs. This is consistent with previous report that ELE increased water holding capacity of meat from hot-stressed broilers and oxidatively stressed pigs [18,19]. Although meat color was not affected by both dietary ELE and pre-slaughter transport in the present study, the tenderness was worse in meat from the T_1 h_ group, which was evidenced by higher (p<0.05) shear force when compared with the two unstressed groups. Pre-slaughter transport increased (p<0.05) the shear force of meat from pigs without ELE supplementation. However, the shear force of meat from T_1 h_+ELE pigs was not higher than that of unstressed pigs, showing that dietary supplementation of ELE eliminated the negative effect of pre-slaughter transport on meat tenderness. In summary, all these data demonstrated that dietary supplementation of ELE improved meat quality and partially eliminated the negative effect of antemortem stress on meat quality.

### Dietary supplementation of ELE inhibited glycolysis in PM muscle

Muscle cells switch to glycolysis for ATP generation after animals are slaughtered. Glycolysis determines the rate and degree of pH decline in PM muscle, which plays a key in the conversion of muscle to meat and meat quality development. As shown in Figure 1A, the GP in muscle from both ELE and T_1 h_+ELE pigs was significantly lower (p<0.05) than that of the other two groups, showing that dietary supplementation of ELE altered carbohydrate metabolism and reduced the substrate for glycolysis in porcine muscle. Muscle pH of the transported pigs was lower (p<0.05) at both 0 and 0.75 h PM than of the unstressed pigs. However, the ultimate muscle pH (pH_24 h_) of the T_1 h_+ELE pigs was not different to that of the unstressed pigs (Figure 1B). Consistently, lactate analysis showed that T_1 h_ pigs had highest (p<0.05) concentrations of lactate in muscle at both 0.75 and 24 h PM (Figure 1C). Muscles from ELE pigs had the lowest lactate at 0.75 h. The concentrations of lactate in muscle from control and T_1 h_+ELE pigs were not different at 0.75 h PM, which were lower than that of T_1 h_ pigs but higher (p<0.05) than that of ELE pigs. In addition, the lactate content in muscle from control, ELE and T_1 h_+ELE pigs were all lower (p<0.05) at 24 h PM when compared to that of the T_1 h_ pigs, which were not different between each other. In summary, all these data showed that dietary supplementation of ELE reduced GP and down-regulated glycolysis in PM porcine muscle, which should contribute to the improve meat quality (Table 4).

For understanding the altered fatty acid profile and carbohydrate metabolism in porcine LT muscle, the activities of some glycogenolytic/glycolytic and oxidative enzymes were analyzed. Although the PK activity was not determined to be different between PM muscles of different pH values (Figure 1E), the activity of GPa was higher (p<0.05) at 0 h PM in the muscle from T_1 h_ pigs (Figure 1D). GPa is the key enzyme in glycogenolysis and controls the production of substrate for glycolysis. The higher GPa activity in T_1 h_ muscle at 0 h PM showed that pre-slaughter transport activated GPa, which is also supported by the higher GPa activities at 0.75 h PM in both groups of transported pigs. However, GPa activity in the muscle from T_1 h_+ELE pigs was not higher than in muscles from unstressed pigs at 0 h PM, indicating that dietary ELE supplementation might alter muscle metabolic response to antemortem stress. CS is the first enzyme of TCA cycle to catalyze the irreversible condensation of acetyle-CoA with oxaloacetate to form citrate. The activities of CS increased (p<0.05) in the muscle from pigs supplemented with ELE at both 0 and 24 h PM when compared with the control (Figure 1F). As the enzyme activities measured at 0 h PM represent the *in vivo* activities, these data show that dietary ELE supplementation increased CS activities and likely the oxidative metabolism capacity of porcine muscle. In addition, CS activity in T_1 h_ muscle was lower (p<0.05) than in the control and T_1 h_+ELE muscle at 0 h PM. Taken with the significantly reduced total fatty acid content in the T_1 h_+ELE muscle, it may be deduced that dietary ELE supplementation altered muscle metabolic response to pre-slaughter transport. Pigs supplemented with ELE relied more on fatty acids and oxidative phosphorylation for energy production in response to stress.

### Dietary ELE supplementation altered metabolite profile in porcine muscle

To better explore the influence of dietary ELE on metabolism, an untargeted metabolomics analysis of porcine LT muscle were conducted using UHPLC-MS/MS. Totally, 659 metabolites were identified in positive mode and 705 in negative mode. As 143 metabolites were identified in both modes, a total of 1,221 distinct metabolites was identified and subjected to MetaboAnalyst analysis in the study.

**Figure 2:**
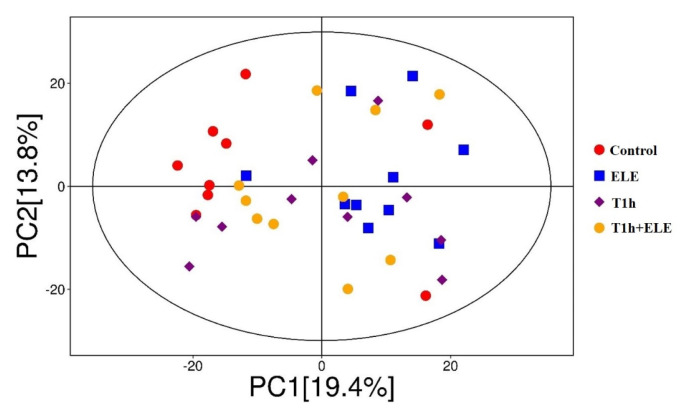
Principal component analysis (PCA) score plots of metabolites in porcine Longissimus thoracis muscle.

As shown in Figure 2, principal component analysis (PCA) of metabolites effectively separated the control from ELE muscle based on PC1, with most muscle samples of the control group located on the left and nine out of ten samples of the ELE group located on the right, which indicates that dietary ELE supplementation induced significant metabolite alteration in porcine muscle. In addition, partial least squares discrimination analysis (PLS-DA) of metabolites effectively separated the muscle samples in pairwise comparison, showing that both dietary ELE supplementation and pre-slaughter transport had an obvious influence on muscle metabolism (Supplementary Figure S1). The differential metabolites between diet groups were filtered with thresholds variable importance in projection (VIP) >1.0 and p<0.05, and visually summarized in volcano plots (Supplementary Figure S2). A full list of the determined differential metabolites was in Table 5 and Supplementary Table S2–7. Most of the differential metabolites are carbohydrate, lipids and lipid-like molecules, amino acids and derivatives, nucleosides, nucleotides, and analogues.

Previous study shows that chronic administration of Eucommia leaf enhances metabolic function of rats across several organs to exert anti-obesity effects under high-fat diet [24]. In agreement, here we found that dietary ELE supplementation to pigs altered metabolism of energy, carbohydrate, lipids, amino acids and so on in porcine muscle. As shown in Table 5, dietary ELE supplementation increased the abundance of phosphocreatine, uridine triphosphate and deoxyguanosine triphosphate, but decreased adenosine diphosphate, creatine, uridine monophosphate, cytidine monophosphate, uridine, and guanosine in porcine muscle, showing that dietary ELE increased energy storage within muscle cells. Fructose 1,6-bisphosphate is an important intermediate in the glycolytic pathway, which is cleaved into two triose phosphates, glyceraldehyde 3-phosphate and dihydroxyacetone phosphate, and ultimately converted to pyruvate. The increased abundance of fructose 1,6-bisphosphate, along with decreased pyruvate and lactate, demonstrated that dietary ELE down-regulated glycolysis in porcine muscle, which agrees with PM muscle pH decline and lactate accumulation (Figure 1B–C) and should account for the enhance water holding capacity of meat (Table 4). Glycolytic analysis revealed that dietary ELE reduced GP within porcine muscle (Figure 1A). Metabolomics analysis by LC-MS/MS determined decreased fructose in muscle from ELE pigs. These data together demonstrated that dietary ELE supplementation to pigs reduced the substrate for glycolysis in muscle. L-acetylcarnitine is a short-chain ester of carnitine that facilitates the influx and efflux of acetyl groups across the mitochondrial inner membrane. The increased abundance of L-acetylcarnitine in the muscle from ELE pigs suggested that dietary ELE improved muscle oxidative metabolism and energy levels [25]. Chronic administration of Eucommia leaf to rats accelerates fatty acid oxidation and increases the use of ketone bodies. This should be the reason for the increased L-acetylcarnitine, glyceric acid, and decreased 3-hydroxybutyric acid, a ketone body, in the LT muscle from pigs supplemented with ELE. Dietary supplementation of ELE also resulted in differential amino acid derivatives, including alanyl-valine, alanyl-isoleucine, 3-methylhistidine, valyl-phenylalanine, valyl-tyrosine, phenylalanyl-glycine, isoleucyl-isoleucine, methionyl-phenylalanine, isoleucyl-phenylalanine, and valyl-methionine.

To better understand the metabolism alteration in porcine muscle induced by dietary ELE, pathway enrichment analysis was performed using differentially expressed metabolites. As shown in Table 6, the Kyoto encyclopedia of genes and genomes (KEGG) enrichment analysis predicted sixteen pathways, including AMPK signaling pathway, carbohydrate metabolism pathways, amino acid metabolism pathways, pyrimidine and purine metabolism pathways, and glucagon signaling pathway. AMPK is the master regulator of cellular metabolism, which regulates glucose, lipid, and protein metabolism [26]. Gain of function in AMPK increases mitochondrial biogenesis and cellular oxidative metabolism capacity [27]. Some studies reports that *Eucommia ulmoides* activates AMPK in rat liver. It is likely that ELE activated AMPK in porcine muscle and thus induced metabolism alteration, which then improves meat quality and alleviated the negative effects of pre-slaughter transport on meat quality (Table 4).

## CONCLUSION

In conclusion, dietary supplementation of 0.5% ELE to growing-finishing pigs had no effect on the proximate composition of porcine muscle but changed muscle FAA and fatty acid profiles. Meat quality, glycolytic, enzymatic and metabolomics analyses revealed that dietary supplementation of ELE improved meat quality and partially eliminated the negative effect of pre-slaughter transport on meat quality by enhancing muscle oxidative metabolism capacity and inhibiting glycolysis in PM muscle, which is probably involved its regulation of AMPK. Our study may provide an effective nutritional strategy to improve pork quality.

## Figures and Tables

**Table 1:** Chemical composition of LT muscle from pigs supplemented with or without *Eucommia ulmoides* leaf extract

Items (%)	Treatments				SEM	Significance		
	
	Control	ELE	T_1 h_	T_1 h_+ELE		Diet	Transport	Diet × transport
Moisture	71.53^a^	71.96^a^	70.81^b^	71.07^b^	0.13	n.s.	*	n.s.
Proteins	22.33	23.66	22.89	23.49	0.33	n.s.	n.s.	n.s.
Fat	2.15	2.16	2.26	2.15	0.03	n.s.	n.s.	n.s.
Ash	1.11^b^	1.13^b^	1.11^b^	1.23^a^	0.01	***	*	*

LT, *Longissimus thoracis*; ELE, *Eucommia ulmoides* leaf extract; SEM, standard error of the mean; n.s., not significant.

* p<0.05;

*** p<0.001.

a,b Within a row, means lacking a common letter differ at p<0.05, n = 10.

**Table 2:** Free amino acid concentrations (mg/100 g muscle) in LT muscle from pigs supplemented with or without ELE

Amino acids	Treatments				SEM	Significance		
	
	Control	ELE	T_1 h_	T_1 h_+ELE		Diet	Transport	Diet × transport
Asp	0.61	0.61	0.56	0.55	0.01	n.s.	n.s.	n.s.
Glu	9.42	7.84	8.75	8.84	0.24	n.s.	n.s.	n.s.
Asn	1.17	1.30	1.11	1.17	0.04	n.s.	n.s.	n.s.
Ser	3.17	3.22	3.05	3.02	0.06	n.s.	n.s.	n.s.
Gln	11.46	12.01	10.64	12.35	0.34	n.s.	n.s.	n.s.
Gly	6.92	7.64	7.27	7.02	0.24	n.s.	n.s.	n.s.
Thr	3.16	2.99	3.07	3.17	0.09	n.s.	n.s.	n.s.
Ala	222.40^b^	243.54^a^	223.50^b^	229.44^ab^	3.21	*	n.s.	n.s.
Tyr	2.38	2.55	2.20	2.28	0.06	n.s.	n.s.	n.s.
Cys	5.43	7.52	6.57	7.05	0.42	n.s.	n.s.	n.s.
Val	3.31	3.33	3.23	3.46	0.07	n.s.	n.s.	n.s.
Trp	0.95	0.88	1.10	1.11	0.05	n.s.	n.s.	n.s.
Ile	4.58	4.85	4.73	4.90	0.10	n.s.	n.s.	n.s.
Leu	4.56	4.81	4.52	4.61	0.12	n.s.	n.s.	n.s.
Lys	4.05	3.68	4.31	3.94	0.16	n.s.	n.s.	n.s.
Arg	2.03^a^	1.86^ab^	1.87^ab^	1.78^b^	0.05	n.s.	n.s.	n.s.
Hyp	39.29^b^	40.51^b^	41.47^ab^	42.37^a^	0.36	n.s.	*	n.s.
Met	2.55^a^	2.56^a^	2.13^b^	2.26^b^	0.06	n.s.	*	n.s.
Phe	7.99^b^	9.21^a^	8.36^ab^	8.76^a^	0.15	*	n.s.	n.s.
Pro	2.82^b^	3.68^a^	3.04^ab^	3.12^ab^	0.11	*	n.s.	n.s.
Sar	9.63^ab^	10.15^a^	9.50^b^	9.80^ab^	0.10	*	n.s.	n.s.
Cit	0.76^b^	0.95 ^a^	0.76^b^	0.91^a^	0.03	*	n.s.	n.s.
Nva	103.40^b^	112.45^a^	103.63^b^	110.20^ab^	1.29	*	n.s.	n.s.
Total	452.04^b^	488.13^a^	455.35^b^	472.10^ab^	4.81	*	n.s.	n.s.

LT, *Longissimus thoracis*; ELE, *Eucommia ulmoides* leaf extract; SEM, standard error of the mean; n.s., not significant.

* p<0.05.

a,b Within a row, means lacking a common letter differ at p<0.05, n = 10.

**Table 3:** Fatty acid concentrations (mg/100 g muscle) in the total lipid fraction of intramuscular fat from LT muscle of pigs supplemented with or without ELE

Fatty acids	Treatments				SEM	Significance		
	
	Control	ELE	T_1 h_	T_1 h_+ELE		Diet	Transport	Diet × transport
C10:0	0.23^b^	0.28^a^	0.19^b^	0.20^b^	0.01	n.s.	*	n.s.
C14:0	2.69^a^	2.81^a^	2.68^a^	2.08^b^	0.09	n.s.	*	n.s.
C16:0	40.07^a^	36.35^a^	38.38^a^	29.61^b^	0.96	***	*	n.s.
C16:1, n7c	5.98^a^	5.62^ab^	6.21^a^	4.60^b^	0.20	*	n.s.	n.s.
C16:1, n9c	0.54^a^	0.50^a^	0.55^a^	0.37^b^	0.02	*	n.s.	n.s.
C17:0	0.28^a^	0.23^bc^	0.26^ab^	0.19^c^	0.01	*	n.s.	n.s.
C17:1, n7c	0.31	0.29	0.32	0.26	0.01	n.s.	n.s.	n.s.
C18:0	20.19^a^	17.44^a^	18.30^a^	13.60^b^	0.53	*	*	n.s.
C18:1, n7c	0.30^a^	0.28^a^	0.32^a^	0.18^b^	0.02	*	n.s.	n.s.
C18:1, n9c	58.57^a^	48.67^b^	56.14^a^	41.63^b^	1.49	***	n.s.	n.s.
C18:1, n9t	5.99^a^	5.30^b^	5.95^ab^	4.22^c^	0.17	*	n.s.	n.s.
C18:2, n6c	18.42^a^	18.27^a^	17.72^a^	10.72^b^	0.62	*	*	*
C20:0	0.29^a^	0.26^a^	0.29^a^	0.19^b^	0.01	*	n.s.	n.s.
C20:1, n9c	1.02	0.75	1.03	0.77	0.05	n.s.	n.s.	n.s.
C20:2, n6c	1.65	1.46	1.64	1.40	0.06	n.s.	n.s.	n.s.
C20:3, n6c	0.53^a^	0.57^a^	0.51^a^	0.35^b^	0.03	n.s.	*	*
C20:4, n6c	2.96^a^	3.12^a^	2.70^a^	1.40^b^	0.13	*	***	*
C20:5, n3c	0.18^b^	0.27^a^	0.18^b^	0.09^c^	0.01	n.s.	*	***
Total	160.98^a^	142.46^a^	153.35^a^	111.83^b^	4.05	***	*	n.s.
SFA^1)^	64.55^a^	57.37^a^	60.10^a^	45.88^b^	1.56	*	*	n.s.
MUFA^2)^	72.70^a^	61.41^b^	70.52^a^	52.03^b^	1.88	***	n.s.	n.s.
PUFA^3)^	23.73^a^	23.68^a^	22.74^a^	13.93^b^	0.81	*	*	*
PUFA/SFA	0.37^a^	0.41^a^	0.38^a^	0.29^b^	0.01	n.s.	*	***
Σn-3^4)^	0.18^b^	0.27^a^	0.18^b^	0.09^c^	0.01	n.s.	*	***
Σn-6^5)^	23.56^a^	23.41^a^	22.56^a^	13.84^b^	0.80	*	*	*
Σn-6/Σn-3	118.38^a^	91.14^b^	133.53^a^	147.93^a^	5.45	n.s.	*	n.s.

LT, *Longissimus thoracis*; ELE, *Eucommia ulmoides* leaf extract; SEM, standard error of the mean; n.s., not significant; SFA, saturated fatty acids; MUFA, monounsaturated fatty acids; PUFA, polyunsaturated fatty acids.

1) SFA, sum of C10:0, C14:0, C16:0, C17:0 C18:0, and C20:0.

2) MUFA, sum of C16:1, C17:1, C18:1, and C20:1.

3) PUFA, sum of C18:2, C20:2, C20:3, C20:4, and C20:5.

4) Σn-3 = C20:5n-3

5) Σn-6 = sum of C18:2n-6, C20:2n-6, and C20:3n-6, and C20:4n-6.

a–c Within a row, means lacking a common letter differ at p<0.05, n = 10.

* p<0.05;

*** p<0.001.

**Table 4:** Meat quality analysis of LT muscle from pigs supplemented with or without ELE

Items	Treatments				SEM	Significance		
	
	Control	ELE	T_1 h_	T_1 h_+ELE		Diet	Transport	Diet × transport
L^*^	44.38	44.71	45.19	45.14	0.31	n.s.	n.s.	n.s.
a^*^	5.78	5.85	5.33	5.86	0.12	n.s.	n.s.	n.s.
b^*^	4.85	4.73	4.26	4.93	0.13	n.s.	n.s.	n.s.
Drip loss (%)	3.99^b^	2.94^c^	4.94^a^	5.10^a^	0.22	n.s.	^***^	n.s.
Cooking yield (%)	78.17^ab^	79.71^a^	76.69^b^	79.33^a^	0.55	^*^.	n.s.	n.s.
Shear force (N)	47.40^b^	47.42^b^	58.55^a^	51.25^ab^	1.62	n.s.	^*^	n.s.

LT, *Longissimus thoracis*; ELE, *Eucommia ulmoides* leaf extract; SEM, standard error of the mean; n.s., not significant.

* p<0.05;

*** p<0.001.

a–c Within a row, means lacking a common letter differ at p<0.05, n = 10.

**Table 5:** Significantly altered metabolites in LT muscle from pigs supplemented with ELE in comparison with the control diet

Metabolites	RT	M/Z	VIP value	p-value	Fold change	Trends
ADP	263.77	428.04	2.06	1.82E-02	4.60E-01	↓
Phosphocreatine	256.41	212.04	1.75	9.49E-03	3.89E+01	↑
Creatine	249.64	132.08	1.27	4.53E-02	8.72E-01	↓
Fructose 1,6-bisphosphate	283.42	338.99	1.81	3.35E-03	3.86E+00	↑
Pyruvic acid	56.06	87.01	1.61	4.32E-02	8.38E-01	↓
L-Lactic acid	136.12	89.02	1.55	3.57E-02	9.39E-01	↓
Leucinic acid	48.75	131.07	1.58	3.21E-02	1.43E+00	↑
Glyceric acid	186.26	105.02	1.62	3.16E-02	7.74E-01	↓
D-Fructose	136.10	179.06	1.42	2.53E-02	7.88E-01	↓
L-Acetylcarnitine	203.47	204.12	1.51	2.33E-02	1.24E+00	↑
(R)-3-Hydroxybutyric acid	143.02	103.04	2.01	1.67E-03	6.72E-01	↓
Guanosine	158.94	284.10	1.91	5.35E-03	6.43E-01	↓
Uridine	77.47	243.06	1.77	2.27E-02	5.87E-01	↓
UMP	255.66	323.03	2.17	1.82E-03	2.42E-01	↓
dGTP	275.28	505.99	2.11	1.20E-03	5.23E+00	↑
UTP	279.31	482.96	1.55	6.07E-03	2.12E+00	↑
CMP	263.75	322.04	2.25	2.15E-03	2.25E-01	↓
8-Hydroxy-2′-deoxyguanosine	158.63	282.08	1.82	1.39E-02	6.85E-01	↓
Alanyl-Valine	153.43	189.12	2.27	4.70E-04	6.48E-01	↓
Alanyl-Isoleucine	141.48	203.14	2.21	6.32E-04	5.34E-01	↓
3-Methylhistidine	190.82	170.09	1.86	1.16E-02	1.29E+00	↑
Valyl-Phenylalanine	107.54	265.15	2.24	1.97E-04	5.18E-01	↓
Valyl-Tyrosine	136.77	281.15	2.21	1.21E-03	5.95E-01	↓
Phenylalanyl-Glycine	141.02	223.11	2.28	2.27E-04	5.44E-01	↓
Isoleucyl-Isoleucine	108.32	245.19	2.21	2.01E-03	4.20E-01	↓
Methionyl-Phenylalanine	98.09	297.13	2.53	6.06E-04	1.86E-01	↓
Isoleucyl-Phenylalanine	93.28	279.17	2.15	1.43E-03	5.50E-01	↓
Valyl-Methionine	123.63	249.13	1.96	1.89E-03	5.78E-01	↓
Hippuric acid	111.48	178.05	1.57	2.42E-02	2.51E+00	↑

LT, *Longissimus thoracis*; ELE, *Eucommia ulmoides* leaf extract; RT, retention time; M/Z, mass charge ratio; VIP, variable importance in projection; ADP, adenosine diphosphate; UMP, uridine monophosphate; CMP, cytidine monophosphate.

**Table 6:** Metabolic pathway enrichment analysis based on the significant metabolites between the ELE and control groups

Pathway name	Pathway ID	Compounds Hit	Compounds KEGG ID
AMPK signaling pathway	ssc04152	4	C00008;C00354;C00022; C04677
Glycolysis/Gluconeogenesis	ssc00010	2	C00186;C00022
Carbon metabolism	ssc01200	4	C00354;C00022;C00258;C00198
Central carbon metabolism in cancer	ssc05230	3	C00354;C00186;C00022
Pentose phosphate pathway	ssc00030	3	C00022;C00258;C00198
Fructose and mannose metabolism	ssc00051	3	C00186;C01094;C03267
Pentose and glucuronate interconversions	ssc00040	2	C00022;C00167
Biosynthesis of amino acids	ssc01230	4	C00327;C00022;C00666;C04916
Glycine, serine and threonine metabolism	ssc00260	3	C00022;C00300;C00258
Arginine and proline metabolism	ssc00330	3	C02305;C00022;C00300
Histidine metabolism	ssc00340	3	C01152;C04677;C04916
Pyrimidine metabolism	ssc00240	6	C00299;C00105;C00075;C00055;C00475;C00380
Purine metabolism	ssc00230	6	C00130;C00008;C00387;C00286;C04677;C03794
Biosynthesis of cofactors	ssc01240	7	C00130;C00008;C00105;C00022;C00075;C00167;C03794
ABC transporters	ssc02010	3	C00387;C00299;C00475
Glucagon signaling pathway	ssc04922	3	C00354;C00186;C00022

ELE, *Eucommia ulmoides* leaf extract; KEGG, Kyoto encyclopedia of genes and genomes.

## SUPPLEMENTARY MATERIAL

**Supplementary Table S1**. Ingredients and nutritional composition of basic diets

**Supplementary Table S2**. Significantly altered metabolites in LT muscle from pigs supplemented with ELE (ELE) in comparison with the control

**Supplementary Table S3**. Significantly altered metabolites in LT muscle from pigs transported for one hour before slaughter (T_1 h_) in comparison with the control

**Supplementary Table S4**. Significantly altered metabolites in LT muscle from pigs supplemented with ELE and transported for one hour before slaughter (T_1 h_+ELE) in comparison with the control

**Supplementary Table S5**. Significantly altered metabolites in LT muscle from pigs transported for one hour before slaughter (T_1 h_) in comparison with pigs supplemented with ELE (ELE)

**Supplementary Table S6**. Significantly altered metabolites in LT muscle from pigs supplemented with ELE and transported for one hour before slaughter (T_1 h_+ELE) in comparison with pigs supplemented with ELE (ELE)

**Supplementary Table S7**. Significantly altered metabolites in LT muscle from pigs supplemented with ELE and transported for one hour before slaughter (T_1 h_+ELE) in comparison with pigs transported for one hour before slaughter (T_1 h_)

**Supplementary Figure S1**. The PLS-DA score plot of metabolites in porcine LT muscle.

**Supplementary Figure S2**. The Volcano plot of muscle metabolites in pairwise comparison.

## References

[1] Daza A, Mateos A, Rey AI, Ovejero I, Lopez-Bote CJ (2007). Effect of duration of feeding under free-range conditions on production results and carcass and fat quality in Iberian pigs. Meat Sci.

[2] Madeira MS, Pires VM, Alfaia CM (2013). Differential effects of reduced protein diets on fatty acid composition and gene expression in muscle and subcutaneous adipose tissue of Alentejana purebred and Large White x Landrace x Pietrain crossbred pigs. Br J Nutr.

[3] Lee YB, Choi YI (1999). PSE (pale, soft, exudative) pork: The causes and solutions-review. Asian-Australas J Anim Sci.

[4] Dong M, Chen HQ, Zhang YM (2020). Processing properties and improvement of pale, soft, and exudative-like chicken meat: a review. Food Bioproc Tech.

[5] Solomon MB, Van Laack RLJM, Eastridge JS (1998). Biophysical basis of pale, soft, exudative (PSE) pork and poultry muscle: a review.

[6] Huang YZ, Zhou LS, Zhang JJ (2020). A large-scale comparison of meat quality and intramuscular fatty acid composition among three Chinese indigenous pig breeds. Meat Sci.

[7] Njoga EO, Ilo SU, Nwobi OC (2023). Pre-slaughter, slaughter and post-slaughter practices of slaughterhouse workers in Southeast, Nigeria: Animal welfare, meat quality, food safety and public health implications. Plos One.

[8] Shen QW, Means WJ, Thompson SA (2006). Pre-slaughter transport, AMP-activated protein kinase, glycolysis, and quality of pork loin. Meat Sci.

[9] Ding H, Cao A, Li H, Zhao Y, Feng J (2020). Effects of Eucommia ulmoides leaf extracts on growth performance, antioxidant capacity and intestinal function in weaned piglets. J Anim Physiol Anim Nutr (Berl).

[10] Huang W, Yao C, Liu Y (2022). Effects of dietary eucommia ulmoides leaf extract on growth performance, expression of feeding-related genes, activities of digestive enzymes, antioxidant capacity, immunity and cytokines expression of large yellow croaker (Larimichthys crocea) larvae. Br J Nutr.

[11] Yan J, Hu R, Li B (2022). Effect of Eucommia ulmoides leaf extract on growth performance, carcass traits, parameters of oxidative stress, and lipid metabolism in broiler chickens. Front Vet Sci.

[12] Chen D, Wang X, Guo Q (2022). Muscle fatty acids, meat flavor compounds and sensory characteristics of Xiangxi yellow cattle in comparison to Aberdeen Angus. Animals (Basel).

[13] Shen QW, Underwood KR, Means WJ, McCormick RJ, Du M (2007). The halothane gene, energy metabolism, adenosine monophosphate-activated protein kinase, and glycolysis in postmortem pig longissimus dorsi muscle. J Anim Sci.

[14] Jiang S, Quan W, Luo J (2023). Low-protein diets supplemented with glycine improves pig growth performance and meat quality: An untargeted metabolomic analysis. Front Vet Sci.

[15] Zheng J, Duan Y, Yu J (2022). Effects of long-term protein restriction on meat quality and muscle metabolites of Shaziling pigs. Animals (Basel).

[16] Park SA, Choi MS, Jung UJ (2006). Eucommia ulmoides Oliver leaf extract increases endogenous antioxidant activity in type 2 diabetic mice. J Med Food.

[17] Liu H, Li K, Zhao J, Deng W (2018). Effects of polyphenolic extract from Eucommia ulmoides Oliver leaf on growth performance, digestibility, rumen fermentation and antioxidant status of fattening lambs. Anim Sci J.

[18] Li H, Zhao J, Deng W, Li K, Liu H (2020). Effects of chlorogenic acid-enriched extract from Eucommia ulmoides Oliver leaf on growth performance and quality and oxidative status of meat in finishing pigs fed diets containing fresh or oxidized corn oil. J Anim Physiol Anim Nutr (Berl).

[19] Zhao JS, Deng W, Liu HW (2019). Effects of chlorogenic acid-enriched extract from Eucommia ulmoides leaf on performance, meat quality, oxidative stability, and fatty acid profile of meat in heat-stressed broilers. Poult Sci.

[20] Li Y, Sato T, Metori K, Koike K, Che Q, Takahashi S (1998). The promoting effects of geniposidic acid and aucubin in Eucommia ulmoides Oliver leaves on collagen synthesis. Biol Pharm Bull.

[21] Shioya M, Maruyama K, Takahashi S, Tanimoto SY (1996). Myofibrils and meat texture of broilers fed on a diet of tochu leaf powder. Biosci Biotechnol Biochem.

[22] Tanimoto SY, Ikuma K, Takahashi S (1993). Improvement in raw meat texture of cultured eel by feeding of Tochu leaf powder. Biosci Biotechnol Biochem.

[23] Zhao X, Wang Y, Nie Z (2020). Eucommia ulmoides leaf extract alters gut microbiota composition, enhances short-chain fatty acids production, and ameliorates osteoporosis in the senescence-accelerated mouse P6 (SAMP6) model. Food Sci Nutr.

[24] Fujikawa T, Hirata T, Wada A (2010). Chronic administration of Eucommia leaf stimulates metabolic function of rats across several organs. Br J Nutr.

[25] Smeland OB, Meisingset TW, Borges K, Sonnewald U (2012). Chronic acetyl-L-carnitine alters brain energy metabolism and increases noradrenaline and serotonin content in healthy mice. Neurochem Int.

[26] Garcia D, Shaw RJ (2017). AMPK: Mechanisms of cellular energy sensing and restoration of metabolic balance. Mol Cell.

[27] Garcia-Roves PM, Osler ME, Holmstrom MH, Zierath JR (2008). Gain-of-function R225Q mutation in AMP-activated protein kinase gamma3 subunit increases mitochondrial biogenesis in glycolytic skeletal muscle. J Biol Chem.

