# Supplementary material for: Dietary supplementation of Eucommia leaf extract to growing-finishing pigs alters muscle metabolism and improves meat quality
Source: Anim Biosci. 2023 Nov 1;37(4):697–708. doi: 10.5713/ab.23.0220 (PMC10915222; doi:10.5713/ab.23.0220)
Supplement: Supplementary file 1 [file ab-23-0220-Supplementary-Table-S1.pdf]

Table S1. Ingredients and nutritional composition of basic diets

Ingredients (%)	Dietary treatment		
	10-30 kg	30-70 kg	70-115 kg
Corn	63.70	58.60	67.00
Soybean meal	19.80	29.00	23.76
Dried whey	4.30	-	-
Wheat bran	-	7.80	6.00
Fish meal	9.00	-	-
Soybean oil	0.80	1.55	0.88
Lys	0.38	0.18	0.01
Met	0.10	0.00	0.00
Thr	0.09	0.01	0.00
Trp	0.01	0.00	0.00
$CaHPO_4$	0.00	0.69	0.50
Limestone	0.52	0.87	0.55
Salt	0.30	0.30	0.30
Premix	1.00	1.00	1.00
Total	100.00	100.00	100.00
Nutrient content (%)			
DE (MJ/kg)	14.60	14.20	14.20
CP	20.27	18.27	16.30
Total Lys	1.52	1.15	0.88
Total (Met + Cys)	0.79	0.61	0.55
Total Thr	0.94	0.77	0.68
Total Trp	0.26	0.25	0.21
Total Ca	0.69	0.60	0.52
Total P	0.57	0.51	0.45
