# Supplementary material for: Dietary supplementation of Eucommia leaf extract to growing-finishing pigs alters muscle metabolism and improves meat quality
Source: Anim Biosci. 2023 Nov 1;37(4):697–708. doi: 10.5713/ab.23.0220 (PMC10915222; doi:10.5713/ab.23.0220)
Supplement: Supplementary file 2 [file ab-23-0220-Supplementary-Table-S2.pdf]

Table S2. Significantly altered metabolites in LT muscle from pigs supplemented with ELE (ELE) in comparison with the control.

Metabolites	RT	M/Z	VIP Value	P-value	Fold change	Trends
Hydrogen phosphate	261.08	96.97	1.36	4.67E-02	8.45E-01	↓
(R)-3-Hydroxybutyric acid	143.02	103.04	2.01	1.67E-03	6.72E-01	↓
Formononetin	127.29	267.07	1.52	4.89E-02	5.71E-01	↓
2-Hydroxy-3-methylbutyric acid	67.44	117.06	1.36	5.00E-02	1.33E+00	↑
L-Acetylcarnitine	203.47	204.12	1.51	2.33E-02	1.24E+00	↑
Inosinic acid	277.92	347.04	2.14	1.84E-03	6.27E-01	↓
Citrulline	229.78	174.09	1.37	4.10E-02	1.28E+00	↑
Alanyl-Valine	153.43	189.12	2.27	4.70E-04	6.48E-01	↓
ADP	263.77	428.04	2.06	1.82E-02	4.60E-01	↓
Phosphocreatine	256.41	212.04	1.75	9.49E-03	3.89E+01	↑
Guanosine	158.94	284.10	1.91	5.35E-03	6.43E-01	↓
Leucinic acid	48.75	131.07	1.58	3.21E-02	1.43E+00	↑
Uridine	77.47	243.06	1.77	2.27E-02	5.87E-01	↓
Fructose 1,6-bisphosphate	283.42	338.99	1.81	3.35E-03	3.86E+00	↑
Uridine 5'-monophosphate	255.66	323.03	2.17	1.82E-03	2.42E-01	↓
Salviaflaside methyl ester	127.73	537.17	1.51	4.84E-02	4.95E-01	↓
8-Hydroxy-2'-deoxyguanosine	158.63	282.08	1.82	1.39E-02	6.85E-01	↓
Hydroxyphenyllactic acid	107.13	181.05	1.67	9.58E-03	7.48E-01	↓
Alanyl-Isoleucine	141.48	203.14	2.21	6.32E-04	5.34E-01	↓
L-Lactic acid	136.12	89.02	1.55	3.57E-02	9.39E-01	↓
3-Methylhistidine	190.82	170.09	1.86	1.16E-02	1.29E+00	↑
3-Indoleacrylic acid	159.40	188.07	1.42	3.97E-02	7.52E-01	↓
Allantoin	98.97	157.04	1.55	9.31E-03	7.39E-01	↓
Pyruvic acid	56.06	87.01	1.61	4.32E-02	8.38E-01	↓

Valyl-Phenylalanine	107.54	265.15	2.24	1.97E-04	5.18E-01	↓
dGTP	275.28	505.99	2.11	1.20E-03	5.23E+00	↑
Hippuric acid	111.48	178.05	1.57	2.42E-02	2.51E+00	↑
Oxytetracycline	230.11	459.13	1.71	4.90E-02	6.16E-01	↓
Uridine triphosphate	279.31	482.96	1.55	6.07E-03	2.12E+00	↑
Valyl-Tyrosine	136.77	281.15	2.21	1.21E-03	5.95E-01	↓
Phenylalanyl-Glycine	141.02	223.11	2.28	2.27E-04	5.44E-01	↓
D-Fructose	136.10	179.06	1.42	2.53E-02	7.88E-01	↓
Isoleucyl-Isoleucine	108.32	245.19	2.21	2.01E-03	4.20E-01	↓
Methionyl-Phenylalanine	98.09	297.13	2.53	6.06E-04	1.86E-01	↓
Pyro-L-glutaminyl-L-glutamine	90.55	258.11	1.58	1.14E-02	7.66E-01	↓
Cytidine monophosphate	263.75	322.04	2.25	2.15E-03	2.25E-01	↓
Uridine diphosphate glucuronic acid	273.66	579.02	1.75	1.97E-03	1.44E+00	↑
4-Hydroxycinnamic acid	10.13	165.05	2.08	3.24E-03	4.89E-01	↓
N(6)-(1,2-dicarboxyethyl)AMP	279.31	464.08	1.48	3.21E-02	4.56E-01	↓
Dihydroprudomenin	265.80	495.15	1.88	2.35E-03	5.38E-01	↓
Isoleucyl-Phenylalanine	93.28	279.17	2.15	1.43E-03	5.50E-01	↓
Creatine	249.64	132.08	1.27	4.53E-02	8.72E-01	↓
Glyceric acid	186.26	105.02	1.62	3.16E-02	7.74E-01	↓
Isoamyl 2-furonpropionate	298.73	211.13	1.91	3.57E-02	1.10E+00	↑
Trigonelline	172.66	138.06	1.65	4.82E-02	1.23E+00	↑
Valyl-Methionine	123.63	249.13	1.96	1.89E-03	5.78E-01	↓
