# Supplementary material for: Dietary supplementation of Eucommia leaf extract to growing-finishing pigs alters muscle metabolism and improves meat quality
Source: Anim Biosci. 2023 Nov 1;37(4):697–708. doi: 10.5713/ab.23.0220 (PMC10915222; doi:10.5713/ab.23.0220)
Supplement: Supplementary file 3 [file ab-23-0220-Supplementary-Table-S3.pdf]

Table S3. Significantly altered metabolites in LT muscle from pigs transported for one hour before slaughter (T_{1h}) in comparison with the control.

Metabolites	RT	M/Z	VIP Value	P-value	Fold change	Trends
(R)-3-Hydroxybutyric acid	143.02	103.04	2.06	1.98E-03	5.50E-01	↓
L-Phenylalanine	158.32	166.09	1.43	3.34E-02	8.84E-01	↓
2-Hydroxybutyric acid	105.75	103.04	2.39	4.12E-05	3.76E-01	↓
Pseudouridine	144.39	243.06	2.19	5.98E-04	7.88E-01	↓
Maleic acid	224.61	115.00	1.78	2.96E-02	1.40E+00	↑
Phenylacetylglycine	105.46	192.07	1.57	3.95E-02	1.56E+00	↑
Guanosine	158.94	284.10	1.92	6.45E-03	5.83E-01	↓
Uridine	77.47	243.06	1.79	3.52E-02	5.69E-01	↓
Decanoylcarnitine	118.96	316.25	1.12	4.32E-02	5.62E-01	↓
3-Hydroxyadipic acid 3,6-lactone	185.79	145.05	1.77	1.97E-02	1.15E+00	↑
8-Hydroxy-2'-deoxyguanosine	158.63	282.08	2.01	4.37E-03	5.82E-01	↓
Hydroxyphenyllactic acid	107.13	181.05	2.10	7.61E-04	6.26E-01	↓
L-Tryptophan	159.29	205.10	2.12	7.02E-04	6.14E-01	↓
stearoyl sphingomyelin	112.22	731.61	2.61	2.16E-06	1.33E+00	↑
Spermidine	175.69	146.17	1.52	4.99E-02	8.14E-01	↓
Glycerophosphocholine	271.48	258.11	1.13	4.60E-02	1.39E+00	↑
Metenamine	175.02	141.11	1.73	2.69E-02	1.04E+00	↑
SM(d18:1/24:1(15Z))	108.94	813.68	1.28	3.65E-02	1.20E+00	↑
3-Indoleacrylic acid	159.40	188.07	2.12	8.68E-04	6.10E-01	↓
2-Ketobutyric acid	116.95	101.02	2.16	3.67E-03	1.95E+00	↑
Allantoin	98.97	157.04	2.29	4.99E-04	6.55E-01	↓
2-Methylbutyroylcarnitine	149.18	246.17	1.83	2.41E-02	5.57E-01	↓
Hippuric acid	111.48	178.05	1.73	2.19E-02	2.60E+00	↑

Uracil	36.81	111.02	1.90	6.36E-03	6.18E-01	↓
L-Proline	187.24	116.07	1.87	3.05E-02	1.42E+00	↑
Pyro-L-glutaminyl-L-glutamine	90.55	258.11	1.51	2.97E-02	7.79E-01	↓
Bergapten	152.92	215.03	1.73	3.56E-02	7.94E-01	↓
D-Ribose	48.11	149.04	1.44	4.19E-02	8.67E-01	↓
S-Adenosylhomocysteine	225.80	385.13	1.32	2.46E-02	6.84E-01	↓
Gluconolactone	46.09	177.04	2.06	5.01E-03	7.42E-01	↓
2-Methyl-1-methylthio-2-butene	187.25	117.07	2.01	2.27E-02	1.54E+00	↑
DL-Tryptophan	159.26	203.08	2.16	4.32E-04	5.96E-01	↓
