# Supplementary material for: Dietary supplementation of Eucommia leaf extract to growing-finishing pigs alters muscle metabolism and improves meat quality
Source: Anim Biosci. 2023 Nov 1;37(4):697–708. doi: 10.5713/ab.23.0220 (PMC10915222; doi:10.5713/ab.23.0220)
Supplement: Supplementary file 4 [file ab-23-0220-Supplementary-Table-S4.pdf]

Table S4. Significantly altered metabolites in LT muscle from pigs supplemented with ELE and transported for one hour before slaughter (T_{1h}+ELE) in comparison with the control.

Metabolites	RT	M/Z	VIP Value	P-value	Fold change	Trends
(R)-3-Hydroxybutyric acid	143.02	103.04	2.54	5.87E-07	4.67E-01	↓
Formononetin	127.29	267.07	1.57	2.69E-02	4.96E-01	↓
L-Phenylalanine	158.32	166.09	1.79	6.05E-03	8.09E-01	↓
2-Hydroxybutyric acid	105.75	103.04	2.47	1.84E-06	3.42E-01	↓
Pseudouridine	144.39	243.06	1.85	2.79E-03	8.12E-01	↓
L-Acetylcarnitine	203.47	204.12	1.35	4.75E-02	1.23E+00	↑
Phenylacetylglycine	105.46	192.07	1.98	6.84E-03	2.04E+00	↑
Dodecanoylcarnitine	112.29	344.28	1.16	4.10E-02	5.99E-01	↓
Diethyl fumarate	158.46	173.08	1.72	3.96E-02	7.42E-01	↓
Inosinic acid	277.92	347.04	1.54	3.07E-02	7.25E-01	↓
Guanosine	158.94	284.10	2.00	2.24E-03	5.28E-01	↓
Pyrrolidine	183.12	72.08	1.38	3.86E-02	8.04E-01	↓
Dimethylglycine	194.39	104.07	1.57	3.79E-02	7.65E-01	↓
Uridine	77.47	243.06	1.85	1.12E-02	5.51E-01	↓
Decanoylcarnitine	118.96	316.25	1.52	1.19E-02	6.00E-01	↓
D-Ribose 5-phosphate	256.44	229.01	1.57	1.69E-02	7.94E-01	↓
L-Arginine	292.44	175.12	1.44	2.87E-02	7.76E-01	↓
N-Acetylhistidine	244.31	198.09	1.45	4.41E-02	1.09E+00	↑
Salviaflaside methyl ester	127.73	537.17	1.62	2.33E-02	4.03E-01	↓
8-Hydroxy-2'-deoxyguanosine	158.63	282.08	2.01	3.18E-03	5.33E-01	↓
Hydroxyphenyllactic acid	107.13	181.05	2.13	1.79E-04	6.21E-01	↓
L-Tryptophan	159.29	205.10	1.96	1.49E-03	5.97E-01	↓
stearoyl sphingomyelin	112.22	731.61	2.26	9.44E-05	1.25E+00	↑
Alanyl-Isoleucine	141.48	203.14	1.56	2.54E-02	7.29E-01	↓
Spermidine	175.69	146.17	1.86	2.13E-02	7.89E-01	↓

Metenamine	175.02	141.11	2.06	1.18E-03	1.06E+00	↑
Methionyl-Leucine	206.83	263.15	1.49	3.44E-02	9.54E-01	↓
Indoxyl sulfate	16.50	212.00	1.32	9.42E-03	1.43E+00	↑
PC(P-18:0/18:3(6Z,9Z,12Z))	20.51	768.59	1.64	1.26E-02	1.27E+00	↑
3-Methylhistidine	190.82	170.09	1.67	1.85E-02	1.28E+00	↑
3-Indoleacrylic acid	159.40	188.07	1.99	1.27E-03	5.86E-01	↓
2-Ketobutyric acid	116.95	101.02	2.13	1.01E-02	1.83E+00	↑
Allantoin	98.97	157.04	2.28	3.36E-05	6.22E-01	↓
2-Methylbutyroylcarnitine	149.18	246.17	2.51	5.11E-05	3.90E-01	↓
Pyruvic acid	56.06	87.01	1.67	8.17E-03	1.14E+00	↑
L-2-Hydroxyglutaric acid	233.48	147.03	1.29	4.69E-02	7.96E-01	↓
Hippuric acid	111.48	178.05	2.21	6.90E-03	4.49E+00	↑
Allopurinol-1-ribonucleoside	154.75	269.09	1.61	2.11E-02	4.84E-01	↓
Uracil	36.81	111.02	1.85	1.80E-03	5.82E-01	↓
L-Proline	187.24	116.07	1.46	4.41E-02	1.37E+00	↑
NADH	237.20	664.11	1.59	1.55E-02	6.03E-01	↓
N-Acetylneuraminic acid	222.54	308.10	1.28	2.84E-02	7.46E-01	↓
Creatinine	85.20	114.07	1.52	1.68E-02	9.08E-01	↓
Pyro-L-glutaminyl-L-glutamine	90.55	258.11	1.55	3.32E-02	8.03E-01	↓
Bergapten	152.92	215.03	1.64	2.09E-02	7.29E-01	↓
D-Ribose	48.11	149.04	1.33	3.18E-02	8.92E-01	↓
Hypotaurine	203.94	108.01	2.01	1.16E-03	5.61E-01	↓
S-Adenosylhomocysteine	225.80	385.13	1.67	8.01E-03	6.84E-01	↓
D-Xylose	153.64	149.04	1.81	6.97E-03	7.88E-01	↓
