# Supplementary material for: Dietary supplementation of Eucommia leaf extract to growing-finishing pigs alters muscle metabolism and improves meat quality
Source: Anim Biosci. 2023 Nov 1;37(4):697–708. doi: 10.5713/ab.23.0220 (PMC10915222; doi:10.5713/ab.23.0220)
Supplement: Supplementary file 5 [file ab-23-0220-Supplementary-Table-S5.pdf]

Table S5. Significantly altered metabolites in LT muscle from pigs transported for one hour before slaughter (T_{1h}) in comparison with pigs supplemented with ELE (ELE).

Metabolites	RT	M/Z	VIP Value	P-value	Fold change	Trends
Adenosine monophosphate	250.29	348.07	1.94	3.16E-02	2.21E+00	↑
2-Hydroxy-3-methylbutyric acid	67.44	117.06	2.51	4.90E-04	5.78E-01	↓
2-Hydroxybutyric acid	105.75	103.04	3.09	2.61E-04	4.48E-01	↓
Pseudouridine	144.39	243.06	2.99	1.30E-04	8.52E-01	↓
L-Leucine	165.17	132.10	1.83	4.33E-02	1.22E+00	↑
Nicotinamide	30.37	123.06	2.19	2.11E-02	1.29E+00	↑
Leucinic acid	48.75	131.07	2.43	1.23E-03	5.77E-01	↓
Decanoylcarnitine	118.96	316.25	1.56	3.35E-02	5.52E-01	↓
L-Tryptophan	159.29	205.10	2.48	4.80E-03	7.96E-01	↓
stearoyl sphingomyelin	112.22	731.61	2.49	2.86E-03	1.21E+00	↑
Alanyl-Isoleucine	141.48	203.14	1.93	2.31E-02	1.40E+00	↑
Phenyllactic acid	42.10	165.06	2.39	3.29E-03	6.52E-01	↓
3-Indoleacrylic acid	159.40	188.07	2.23	1.49E-02	8.12E-01	↓
Isobutyryl-L-carnitine	174.29	232.15	2.01	3.55E-02	8.24E-01	↓
Valyl-Phenylalanine	107.54	265.15	2.70	5.49E-04	1.69E+00	↑
L-2-Hydroxyglutaric acid	233.48	147.03	1.66	3.39E-02	7.90E-01	↓
PC(P-18:1(9Z)/16:0)	93.91	744.59	2.17	3.97E-02	1.72E+00	↑
Valyl-Tyrosine	136.77	281.15	2.80	1.23E-03	1.65E+00	↑
Phenylalanyl-Glycine	141.02	223.11	2.45	1.93E-03	1.48E+00	↑
Isoleucyl-Isoleucine	108.32	245.19	2.20	8.41E-03	1.81E+00	↑
Methionyl-Phenylalanine	98.09	297.13	2.45	2.89E-03	2.84E+00	↑
Uridine diphosphate glucuronic acid	273.66	579.02	2.21	3.29E-02	5.72E-01	↓
N(6)-(1,2-	279.31	464.08	2.30	1.31E-02	2.80E+00	↓

dicarboxyethyl)AMP						
Isoleucyl-Phenylalanine	93.28	279.17	2.24	1.29E-02	1.47E+00	↑
Valyl-Methionine	123.63	249.13	1.95	3.17E-02	1.37E+00	↑
DL-Tryptophan	159.26	203.08	2.48	3.32E-03	7.90E-01	↓
