# Supplementary material for: Dietary supplementation of Eucommia leaf extract to growing-finishing pigs alters muscle metabolism and improves meat quality
Source: Anim Biosci. 2023 Nov 1;37(4):697–708. doi: 10.5713/ab.23.0220 (PMC10915222; doi:10.5713/ab.23.0220)
Supplement: Supplementary file 6 [file ab-23-0220-Supplementary-Table-S6.pdf]

Table S6. Significantly altered metabolites in LT muscle from pigs supplemented with ELE and transported for one hour before slaughter (T_{1h}+ELE) in comparison with pigs supplemented with ELE (ELE).

Metabolites	RT	M/Z	VIP Value	P-value	Fold change	Trends
(R)-3-Hydroxybutyric acid	143.02	103.04	2.89	1.89E-04	6.95E-01	↓
L-Phenylalanine	158.32	166.09	2.29	9.02E-03	8.38E-01	↓
2-Hydroxy-3-methylbutyric acid	67.44	117.06	2.66	2.61E-04	6.00E-01	↓
2-Hydroxybutyric acid	105.75	103.04	3.13	1.67E-05	4.08E-01	↓
Pseudouridine	144.39	243.06	2.41	2.19E-03	8.78E-01	↓
Nicotinamide	30.37	123.06	2.49	1.31E-03	1.46E+00	↑
Phenylacetylglycine	105.46	192.07	2.00	8.75E-03	1.37E+00	↑
Diethyl fumarate	158.46	173.08	2.63	6.12E-04	7.03E-01	↓
Citrulline	229.78	174.09	1.87	1.99E-02	7.08E-01	↓
Phosphorylcholine	269.28	184.07	2.23	1.71E-02	1.28E+00	↑
ADP	263.77	428.04	1.87	3.32E-02	1.66E+00	↑
Citric acid	276.54	191.02	1.81	2.43E-02	1.24E+00	↑
Dimethylglycine	194.39	104.07	1.92	4.97E-02	7.79E-01	↓
Leucinic acid	48.75	131.07	2.38	1.81E-03	6.10E-01	↓
Decanoylcarnitine	118.96	316.25	1.97	3.17E-03	5.89E-01	↓
Uridine 5'-monophosphate	255.66	323.03	2.09	1.54E-02	2.64E+00	↑
Glycerol 3-phosphate	250.99	173.02	1.65	4.97E-02	1.16E+00	↑
L-Tryptophan	159.29	205.10	2.25	1.52E-02	7.73E-01	↓
stearoyl sphingomyelin	112.22	731.61	1.62	4.18E-02	1.13E+00	↑
Alanyl-Isoleucine	141.48	203.14	1.75	2.86E-02	1.36E+00	↑
Phenyllactic acid	42.10	165.06	2.65	5.87E-04	6.74E-01	↓
3-Hydroxyisovaleric acid	97.55	117.06	1.75	2.13E-02	6.93E-01	↓
Indoxyl sulfate	16.50	212.00	1.56	8.66E-03	1.41E+00	↓

3-Indoleacrylic acid	159.40	188.07	2.16	1.75E-02	7.79E-01	↓
2-Ketobutyric acid	116.95	101.02	1.86	4.90E-02	1.27E+00	↑
Isobutyryl-L-carnitine	174.29	232.15	2.87	1.53E-04	6.75E-01	↓
Allantoin	98.97	157.04	1.91	3.83E-02	8.41E-01	↓
2-Methylbutyroylcarnitine	149.18	246.17	2.63	2.57E-03	5.49E-01	↓
Pyruvic acid	56.06	87.01	2.42	3.66E-03	1.36E+00	↑
Valyl-Phenylalanine	107.54	265.15	3.02	2.70E-05	2.02E+00	↑
dGTP	275.28	505.99	1.64	1.91E-02	3.40E-01	↓
L-2-Hydroxyglutaric acid	233.48	147.03	2.00	9.83E-03	7.27E-01	↓
Hippuric acid	111.48	178.05	2.40	4.74E-03	1.79E+00	↑
Deoxycytidine	151.53	226.08	1.72	4.59E-02	7.50E-01	↓
Uridine triphosphate	279.31	482.96	1.38	1.31E-02	5.13E-01	↓
Valyl-Tyrosine	136.77	281.15	2.85	8.50E-04	1.74E+00	↑
Phenylalanyl-Glycine	141.02	223.11	2.51	1.05E-03	1.43E+00	↑
Isoleucyl-Isoleucine	108.32	245.19	2.26	6.14E-03	1.96E+00	↑
Methionyl-Phenylalanine	98.09	297.13	2.29	2.37E-03	2.83E+00	↑
NADH	237.20	664.11	1.84	1.35E-02	5.88E-01	↓
Creatinine	85.20	114.07	1.77	4.22E-02	9.33E-01	↓
Uridine diphosphate glucuronic acid	273.66	579.02	1.64	2.49E-02	7.57E-01	↓
N(6)-(1,2-dicarboxyethyl)AMP	279.31	464.08	1.94	2.02E-02	2.53E+00	↑
