# Supplementary material for: Dietary supplementation of Eucommia leaf extract to growing-finishing pigs alters muscle metabolism and improves meat quality
Source: Anim Biosci. 2023 Nov 1;37(4):697–708. doi: 10.5713/ab.23.0220 (PMC10915222; doi:10.5713/ab.23.0220)
Supplement: Supplementary file 7 [file ab-23-0220-Supplementary-Table-S7.pdf]

Table S7. Significantly altered metabolites in LT muscle from pigs supplemented with ELE and transported for one hour before slaughter (T_{1h}+ELE) in comparison with pigs transported for one hour before slaughter (T_{1h}).

Metabolites	RT	M/Z	VIP Value	P-value	Fold change	Trends
Phenylacetylglycine	105.46	192.07	1.99	4.84E-02	1.31E+00	↑
Pipecolic acid	187.16	130.09	1.84	3.90E-02	7.43E-01	↓
PC(P-16:0/16:0)	43.64	718.57	3.09	5.51E-03	7.26E-01	↓
Isobutyryl-L-carnitine	174.29	232.15	2.58	1.28E-02	8.19E-01	↓
Hippuric acid	111.48	178.05	2.87	9.23E-03	1.73E+00	↑
PC(P-18:1(9Z)/16:0)	93.91	744.59	2.74	1.56E-02	5.26E-01	↓
