# Supplementary figures and images for: Dietary supplementation of Eucommia leaf extract to growing-finishing pigs alters muscle metabolism and improves meat quality
Source: Anim Biosci. 2023 Nov 1;37(4):697–708. doi: 10.5713/ab.23.0220 (PMC10915222; doi:10.5713/ab.23.0220)

**Figure S1.** The PLS-DA score plot of metabolites in porcine LT muscle.

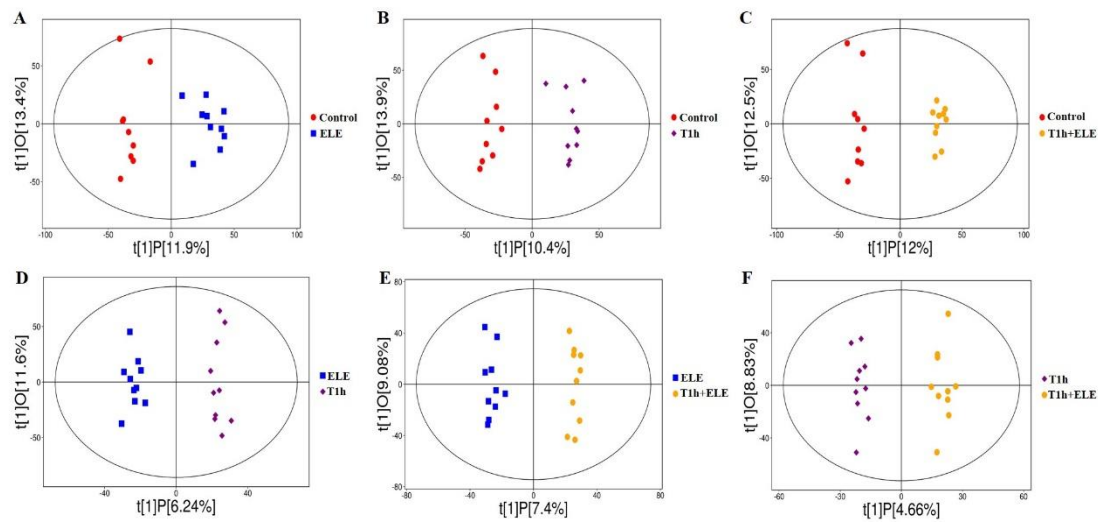

Supplement: Supplementary file 8 [file ab-23-0220-Supplementary-Fig-S1.pdf]
