# Supplementary material for: Dietary supplementation of Eucommia leaf extract to growing-finishing pigs alters muscle metabolism and improves meat quality
Source: Anim Biosci. 2023 Nov 1;37(4):697–708. doi: 10.5713/ab.23.0220 (PMC10915222; doi:10.5713/ab.23.0220)

Figure S2. The Volcano plot of muscle metabolites in pairwise comparison. Metabolites in red were up-regulated and metabolites in blue were down-regulated. Differential metabolites marked in the plot were determined by combining restrictions of $p < 0.05$ and VIP > 1.0.

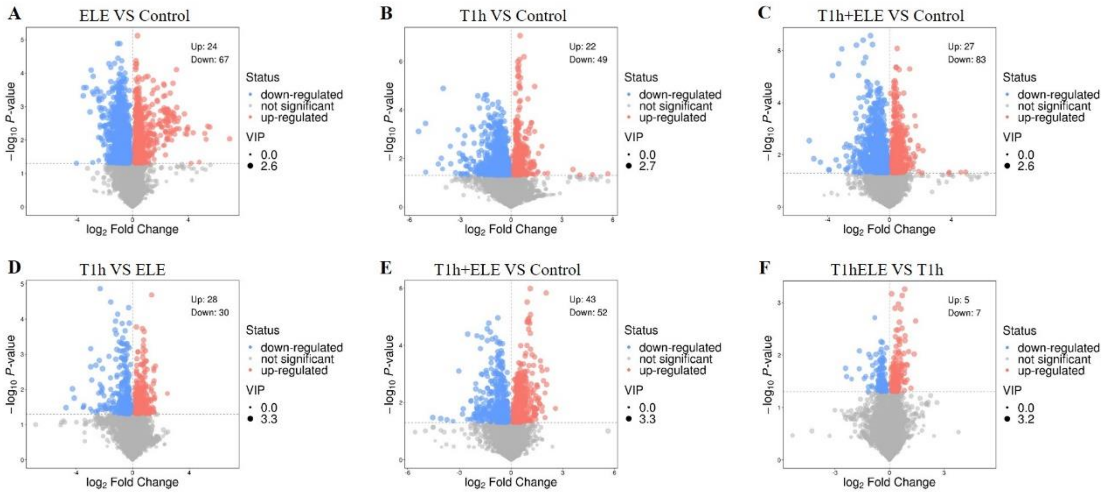

Supplement: Supplementary file 9 [file ab-23-0220-Supplementary-Fig-S2.pdf]
